# Supplementary figures and images for: Network topology metrics explaining enrichment of hybrid epithelial/mesenchymal phenotypes in metastasis
Source: PLoS Comput Biol. 2022 Nov 8;18(11):e1010687. doi: 10.1371/journal.pcbi.1010687 (PMC9674141; doi:10.1371/journal.pcbi.1010687)

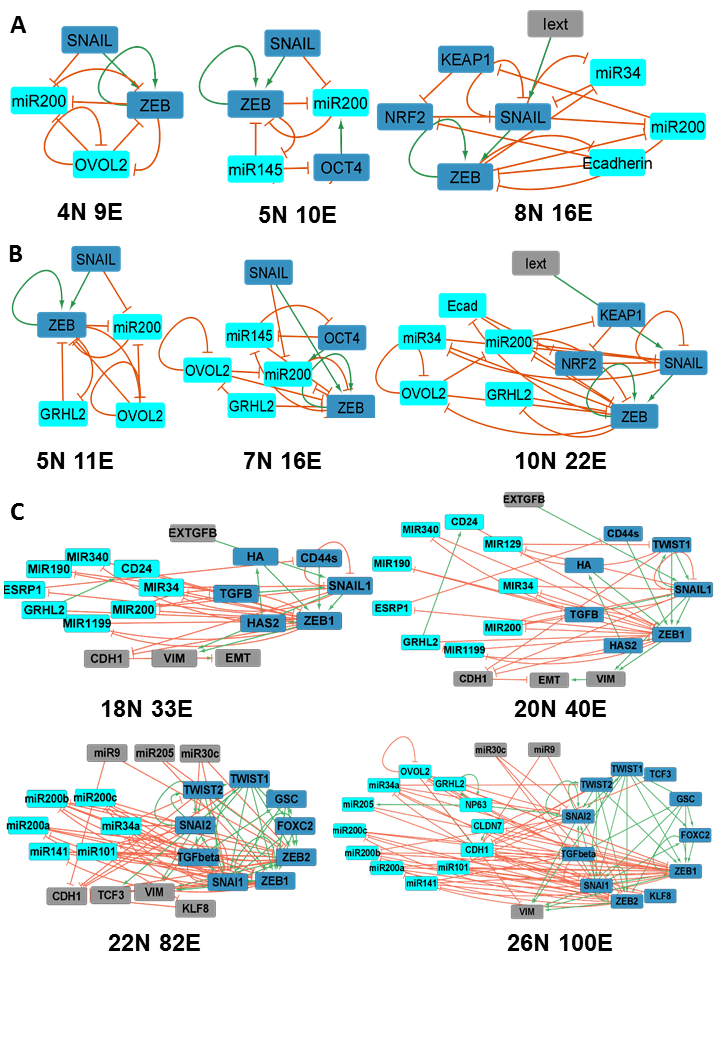

Supplement: S1 Fig — (A) Small size networks. (B) Medium size networks. (C) Large networks. Cyan colored nodes represent epithelial marker genes and blue colored nodes represent mesenchymal marker genes. Red edges represent inhibitions and green edges represent activations. (TIF) [file pcbi.1010687.s001.tif]

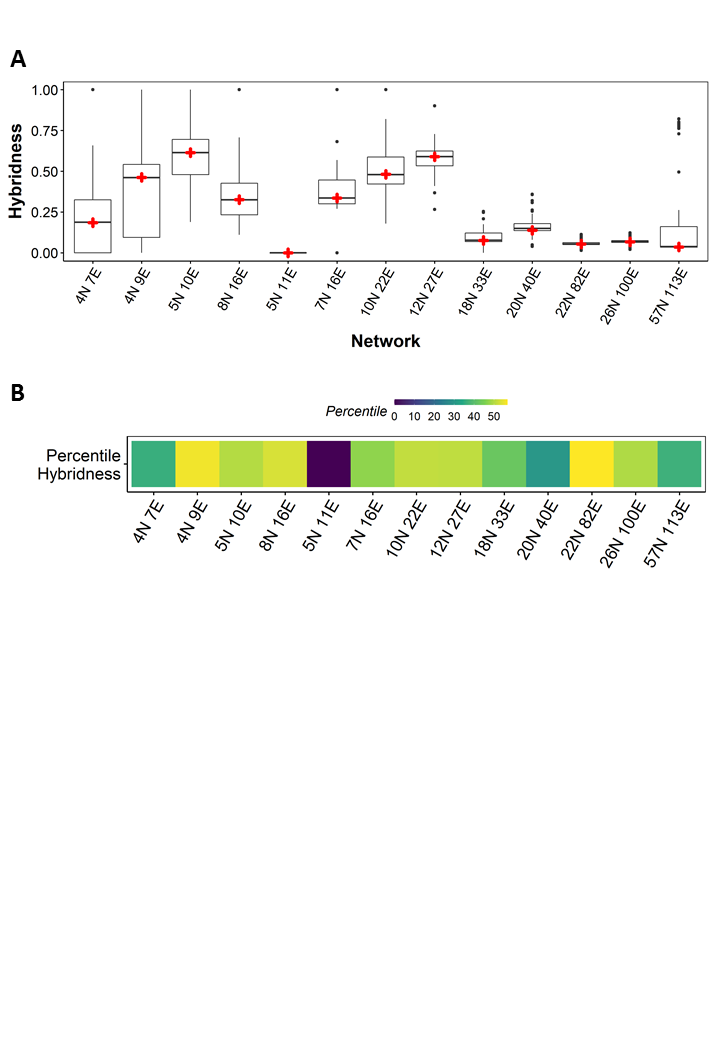

Supplement: S2 Fig — (A) Each network is perturbed 2*E times, where E represents the number of edges, and hybridness of each perturbed network is calculated. Red mark represents hybridness of a WT topology of any network and the associated distribution is the hybridness of all of its perturbations. (B) Percentile hybridness of WT EMP networks in the distributions in S2A. (TIF) [file pcbi.1010687.s002.tif]

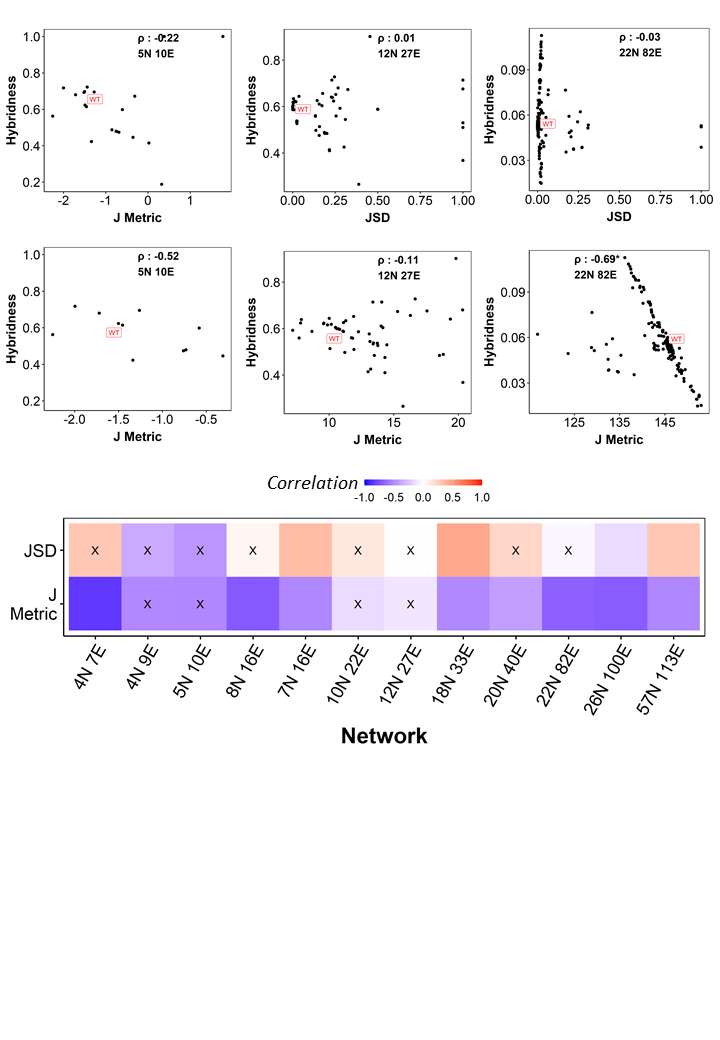

Supplement: S3 Fig — (A) Representative scatter plots between JSD and hybridness. (B) Representative scatter plots between J metric and hybridness. (C) Spearman correlation between hybridness and JSD (top row) and J metric (bottom row) across the networks. “X” represents p-value > 0.05. (TIF) [file pcbi.1010687.s003.tif]

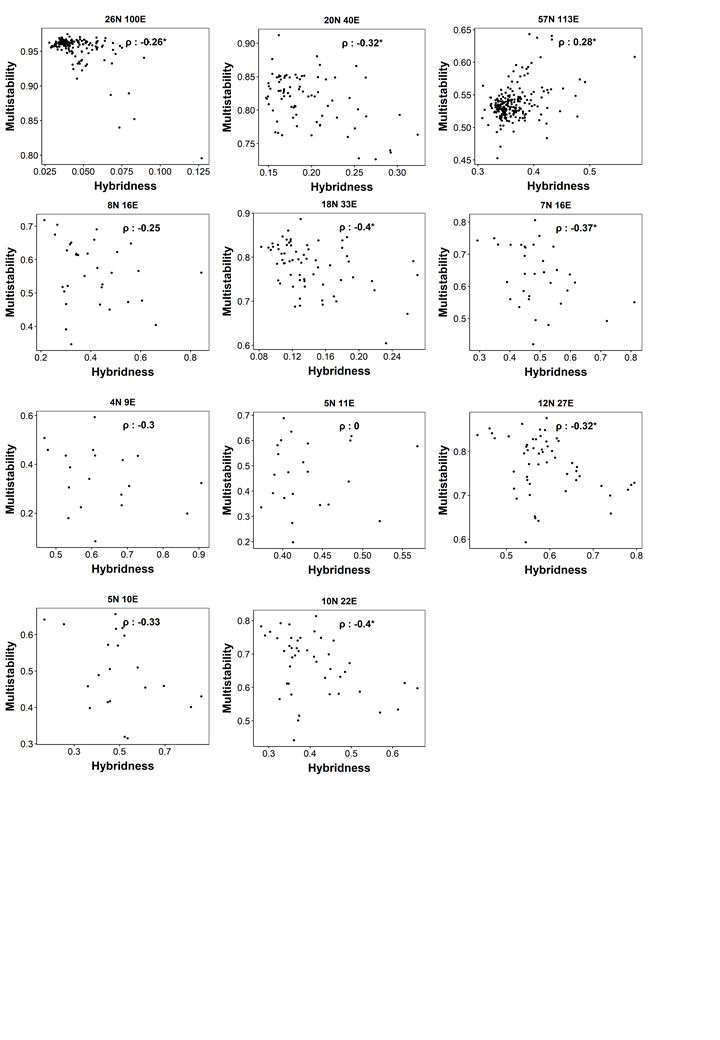

Supplement: S4 Fig — (TIF) [file pcbi.1010687.s004.tif]

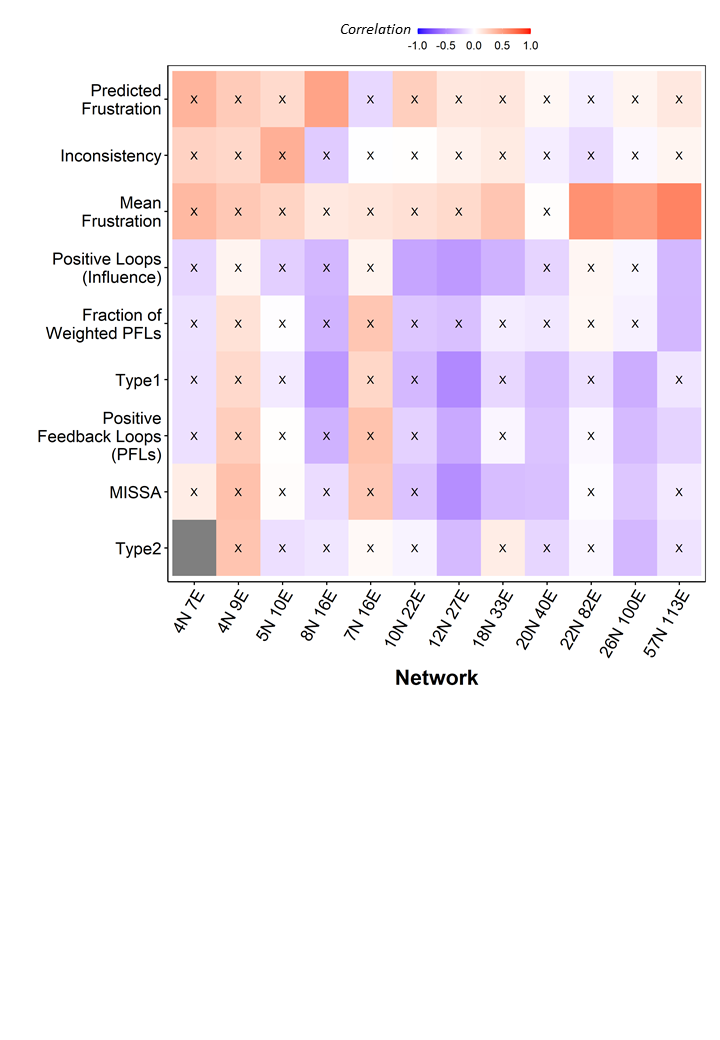

Supplement: S5 Fig — (TIF) [file pcbi.1010687.s005.tif]

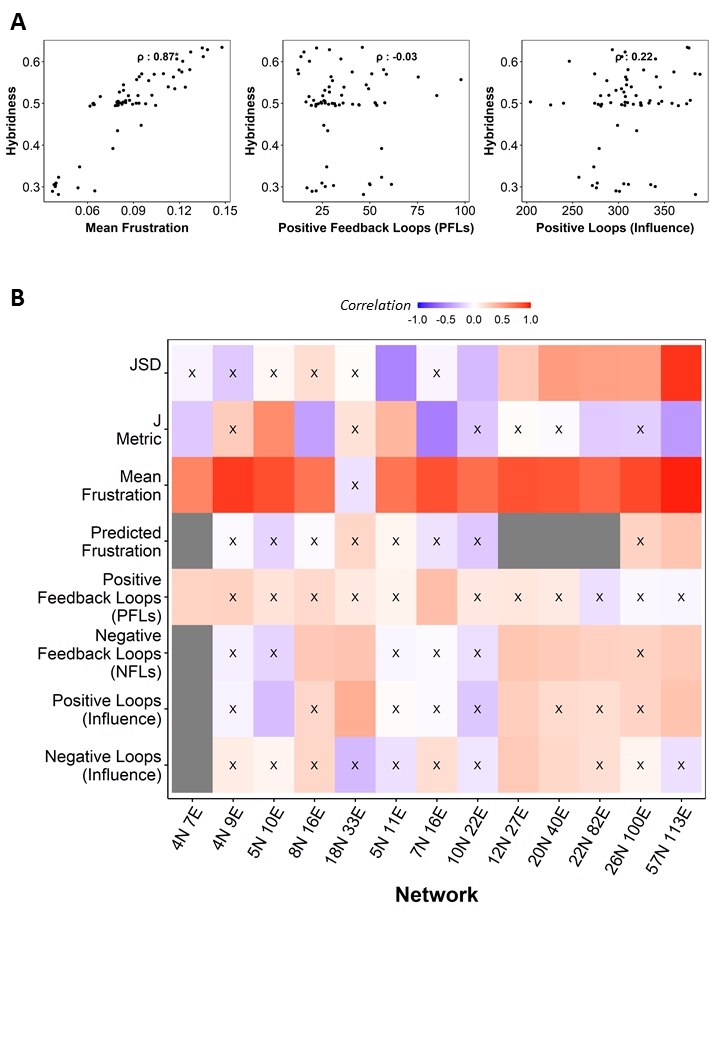

Supplement: S6 Fig — (TIF) [file pcbi.1010687.s006.tif]
