# Supplementary material for: Network topology metrics explaining enrichment of hybrid epithelial/mesenchymal phenotypes in metastasis
Source: PLoS Comput Biol. 2022 Nov 8;18(11):e1010687. doi: 10.1371/journal.pcbi.1010687 (PMC9674141; doi:10.1371/journal.pcbi.1010687)
Supplement: Attachment — Submitted filename: Jolly.pdf [file pcbi.1010687.s008.pdf]

In the manuscript 'Network topology metrics explaining enrichment of hybrid epithelial mesenchymal phenotypes in metastasis', Rashid et al. performed mathematical modeling and network topology analyses to study the influence of network structures on the ability of epithelial-mesenchymal plasticity (EMP) gene networks to generate hybrid cell states. The authors defined 'hybridness' as the extent to which the expressions of E genes and M genes are balanced. Through ODE and Boolean network simulations, the authors found that several EMP networks have low 'hybridness' compared to their perturbed counterparts. Furthermore, the authors found that hybridness was positively correlated with changes of gene expression patterns, and negatively correlated with multistability and numbers of several forms of positive feedback loops, including high-order positive feedback loops. The authors also found that the hybrid phenotypes had high frustration. Overall, the analyses of several EMP gene networks are a welcome addition to the field. The combinations of ODE and Boolean methods for simulations, as well as several network metrics, were thoroughly performed. I have the following comments that are aimed at improving the manuscript.

1. The definition of E and M genes in each EMP model is not very clear. The authors mentioned that 'the Epithelial and Mesenchymal genes in the network are identified through higher order clustering by using hclust function'. I think it is better to state the name 'hierarchical clustering' explicitly in Methods. Did the authors look for two main clusters, one for E and the other for M? Were marker genes used to assign the E and M identities to the two clusters? How accurate is the method in classifying E and M genes that were previously annotated (e.g. doi: 10.15252/emmm.201404208 and doi: 10.1038/s41540-019-0097-0)?

2. The description of the 13 selected gene network models seems to be insufficient. How were the models generated? Were the edges directly derived from experimental data or some inference methods?

3. The authors performed a comprehensive analysis of several structures containing interconnected positive feedback loops. This is a strength of this study. The definitions of Type I and Type II positive feedback loop clusters and their relevance to high-order multistability were first introduced in Ye et al. (doi: 10.1371/journal.pcbi.1006855). I suggest that the authors cite this work as well.

The following 3 comments are regarding the definition of 'hybridness'. As a central concept of this paper, I feel the authors can improve its clarity and build a stronger connection to previous theories and experimental data.

4. If I understand it correctly, the authors defined hybridness as the extent to which the overall E gene expression cancels that of the M gene expression out. This is a reasonable metric, but I think it has some limitations. For example, when the expressions of both E genes and M genes are both low, the metric can give a high hybridness score, but biologically it would be difficult to justify a hybrid phenotype in this scenario. I suggest that the authors discuss some alternative approaches to define hybridness. For example, a scoring system can encourage some expression of both E and M genes. Another possible definition is based on the partial expression of each gene (see Point 4). It is likely that none of these metrics is perfect, but I think giving readers a more unbiased perspective will be helpful.

5. The authors applied their definition of hybridness to many gene network models. While this is useful, it is unclear how one can apply this metric to connect models to experimental data. There is a wealth of published data on progression of EMT in cell lines and EMP-related tumors. It will be helpful to show that the authors' analysis of hybridness can elucidate hybrid cells/samples in data.

6. The authors provided a good discussion on the trade-off between hybridness and multistability. However, some additional reconciliation between the authors' findings on hybridness and previous theories of hybrid E/M states (including the authors' own work doi: 10.1073/pnas.1318192110) seems to be necessary. In previous models, hybrid E/M states occur in conjunction with multistability, but the authors claimed an anticorrelation between hybridness and multistability in the present work. This discrepancy can be confusing to readers if the authors do not provide a clearer explanation.

7. Please fix some minor issued in figures, e.g. the label 'Percentile' overlapped with a number in the color bar of Figure 5E, and an incomplete self-regulation arrow of OVOL2 in Figure S1C.
