# Supplementary material for: Network topology metrics explaining enrichment of hybrid epithelial/mesenchymal phenotypes in metastasis
Source: PLoS Comput Biol. 2022 Nov 8;18(11):e1010687. doi: 10.1371/journal.pcbi.1010687 (PMC9674141; doi:10.1371/journal.pcbi.1010687)
Supplement: Attachment — Submitted filename: Response letter_Oct 6.pdf [file pcbi.1010687.s009.pdf]

(PCOMPBIOL-D-22-00802)

Dear editor, Prof. James R. Faeder

Thank you very much for the letter concerning the manuscript ***"Network Topology Metrics Explaining Enrichment of Hybrid Epithelial Mesenchymal Phenotypes in Metastasis"***. We thank all the reviewers for taking time in reading our manuscript and providing valuable suggestions. We especially thank referee #2 for highlighting many mistakes that improved our manuscript a lot. We have gone through all the comments very carefully and have made the necessary corrections. We hope that the revised version of the manuscript meets the expectations of the reviewers and the journal.

Please find below our response to the comments. The corrections made in the manuscript are highlighted in yellow.

Thank you,

Mubasher
(On behalf of all the co-authors)

Response to reviewer's comments

Reviewer #1:

In the manuscript 'Network topology metrics explaining enrichment of hybrid epithelial-mesenchymal phenotypes in metastasis', Rashid et al. performed mathematical modeling and network topology analyses to study the influence of network structures on the ability of epithelial-mesenchymal plasticity (EMP) gene networks to generate hybrid cell states. The authors defined 'hybridness' as the extent to which the expressions of E genes and M genes are balanced. Through ODE and Boolean network simulations, the authors found that several EMP networks have low 'hybridness' compared to their perturbed counterparts. Furthermore, the authors found that hybridness was positively correlated with changes of gene expression patterns, and negatively correlated with multistability and numbers of several forms of positive feedback loops, including high-order positive feedback loops. The authors also found that the hybrid phenotypes had high frustration. Overall, the analyses of several EMP gene networks are a welcome addition to the field. The combinations of ODE and Boolean methods for simulations, as well as several network metrics, were thoroughly performed. I have the following comments that are aimed at improving the manuscript.

We appreciate the reviewer's positive comments regarding our thorough analyses of EMP networks. Please find our responses to the comments below.

Comment 1

The definition of E and M genes in each EMP model is not very clear. The authors mentioned that 'the Epithelial and Mesenchymal genes in the network are identified through higher order clustering by using hclust function'. I think it is better to state the name 'hierarchical clustering' explicitly in Methods. Did the authors look for two main clusters, one for E and the other for M? Were marker genes used to assign the E and M identities to the two clusters? How accurate is the method in classifying E and M genes that were previously annotated (e.g. doi: 10.15252/emmm.201404208 and doi: 10.1038/s41540-019-0097-0)?

Response:

We have introduced a new section "**Classification of network nodes**" in methods explaining the details of classification. Moreover, a comparison with the annotations in Tan et al. 2014 and Watanabe et al. 2019 shows that the following two sets of genes match the classification: Epithelial(GRHL2, OVOL2, CDH1, miR-200c, CLDN7, GSK3) and Mesenchymal(ZEB1, VIM, TWIST1, ZEB2, SNAI1, SNAI2, TWIST2, KLF8, GSC, TWIST1).

"Classification of network nodes

Given the nature of the networks analysed here, we could classify the nodes (genes) participating in the network into two categories: Epithelial and Mesenchymal. First, we calculated pairwise correlations between the expression values of the nodes obtained from

RACIPE, using the cor function, thus obtaining a pairwise correlation matrix. We then applied hierarchical clustering on the correlation matrix (hclust, distance method Euclidean, agglomeration method complete). We split the resultant dendrogram into two chunks (cutree), resulting in two clusters of nodes. These clusters were then labelled based on the biochemical nature of the composing nodes as Epithelial or Mesenchymal.

Comment 2

The description of the 13 selected gene network models seems to be insufficient. How were the models generated? Were the edges directly derived from experimental data or some inference methods?

Response:

We have now added references to all the models and modified the language as well. The models have been constructed from the experimental data and have previously been reported in the references cited in the updated text below;

*"To understand the emergence and frequency of hybrid phenotypes, we investigated 13 different GRNs of varying sizes. These networks can be categorized into three classes based on their size (number of nodes and edges): (i) four small-sized networks (**Fig 1A i, Fig S1**). (ii) four composite networks formed by combining the small-sized networks (**Fig 1A ii, Fig S1)** and (iii) five large-scale networks (**Fig 1A iii, Fig S1**). We labelled each network with the number of nodes and edges in the network (ex: 4N 7E for the smallest network). The small and large networks have previously been reported to be underlying EMP (Jolly et al. 2016; Boci et al 2019; Silveira and Mombach, 2019; Silveira et al. 2020; Huang et al. 2017; Tripathi et al. 2020a; Font-Clos et al. 2018; Hari et al. 2020; Hari et al. 2021). Given the demonstrated role of these networks in EMP, we constructed the composite networks to check the validity of our results when the assumption that these networks are isolated is relaxed."*

Comment 3

The authors performed a comprehensive analysis of several structures containing interconnected positive feedback loops. This is a strength of this study. The definitions of Type I and Type II positive feedback loop clusters and their relevance to high-order multistability were first introduced in Ye et al. (doi: 10.1371/journal.pcbi.1006855). I suggest that the authors cite this work as well.

Response:

We thank the reviewer for the appreciation. The suggested reference has been included.

The following 3 comments are regarding the definition of 'hybridness'. As a central concept of this paper, I feel the authors can improve its clarity and build a stronger connection to previous theories and experimental data.

<u>Comment 4</u>

If I understand it correctly, the authors defined hybridness as the extent to which the overall E gene expression cancels that of the M gene expression out. This is a reasonable metric, but I think it has some limitations. For example, when the expressions of both E genes and M genes are both low, the metric can give a high hybridness score, but biologically it would be difficult to justify a hybrid phenotype in this scenario. I suggest that the authors discuss some alternative approaches to define hybridness. For example, a scoring system can encourage some expression of both E and M genes. Another possible definition is based on the partial expression of each gene (see Point 4). It is likely that none of these metrics is perfect, but I think giving readers a more unbiased perspective will be helpful.

Response:

Thank you for the suggestion related to comparison of several EMT signatures.

The specific cases where expressions of both E and M genes is low do come up, but with a very low frequency. We quantified the same below (Fig R1). For each state, we individually calculated the E score (the number of E nodes on divided by the total number of E nodes) and M score for each WT network for both Boolean and RACIPE simulations. With the threshold of 0.5 for both scores, we divide the 2-D space defined by E score and M score into four quadrants (Fig R1a). The low-low states would hence fall in quadrant 3. We calculate the fraction of hybrid states in quadrant 3 for all networks, i.e., the sum of frequency of hybrid states in quadrant 3 divided by the total frequency of hybrid states in a given network (Fig R1 b, c). In RACIPE simulations (on which most of our inferences are based), all networks show a hybrid state fraction of less than 0.1. In Boolean, 9 out of the 13 networks show a fraction of 0, one network shows a fraction of 0.1, while the remaining three networks show a larger fraction of hybrid states in quadrant 3.

Further, apart from the reviewers suggestions, there are several other (gene-specific as well as cancer-specific) EMT signatures already there that can be employed to provide an unbiased perspective of hybridness of the EMP networks. However, since we are looking to connect model to the experimental data in a follow up study, we would definitely consider alternate approaches to define hybridness in that study. We would appreciate if the reviewer can bear with us at this stage.

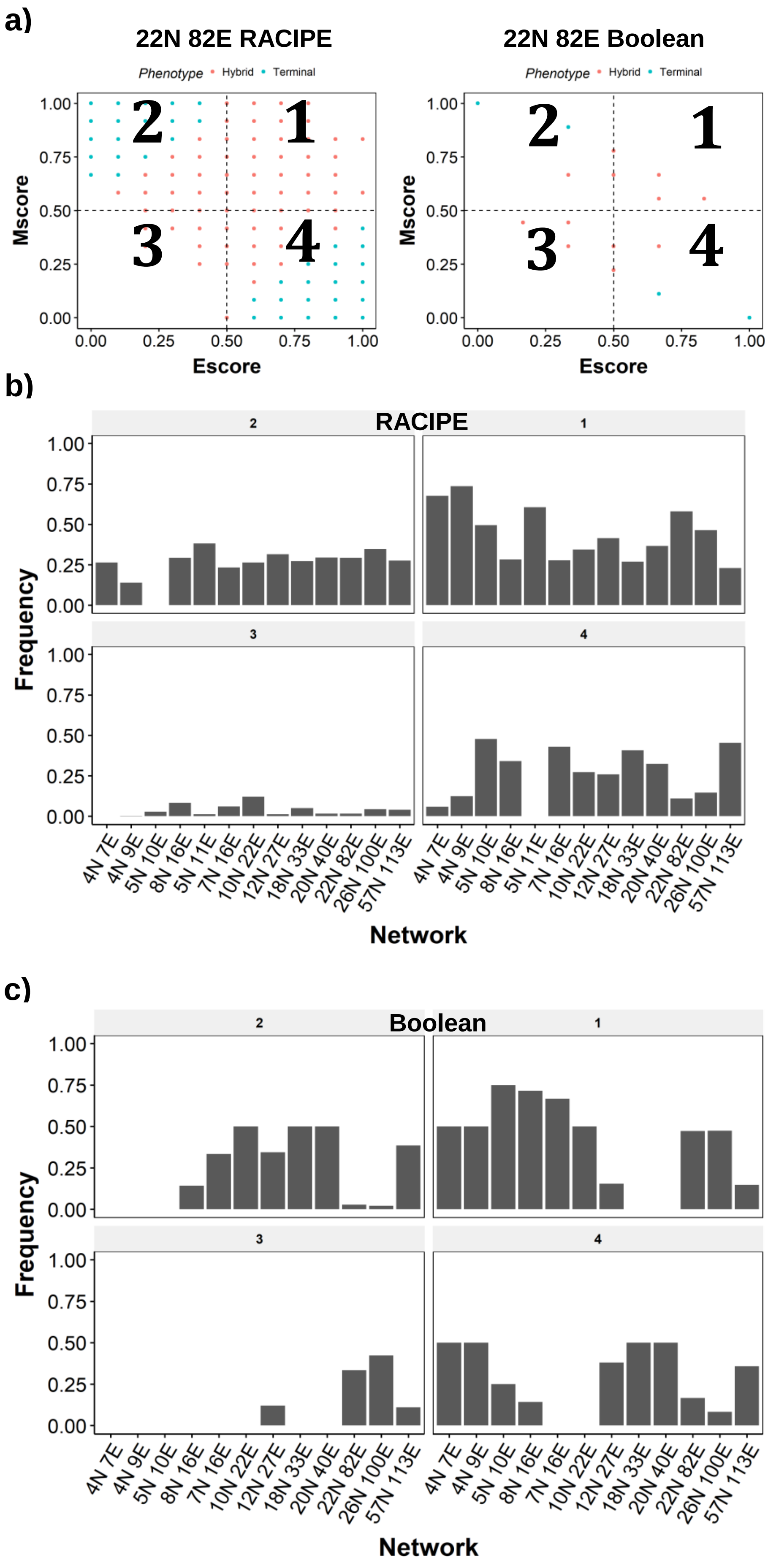
a)
22N 82E RACIPE
22N 82E Boolean
Phenotype • Hybrid • Terminal
Mscore
Escore
1.00
0.75
0.50
0.25
0.00
1
2
3
4
b)
RACIPE
c)
Boolean
Frequency
Network
4N 7E
4N 9E
5N 10E
8N 16E
5N 11E
7N 16E
10N 22E
12N 27E
18N 33E
20N 40E
22N 82E
26N 100E
57N 113E

Figure R1. *a) Depiction of the four quadrants and distribution of states for 22N 82E network for RACIPE (left) and Boolean (right). b) Quantification of the fraction of hybrid states obtained in RACIPE simulations in each quadrant (label on top of each sub-panel). c) Same as b but for Boolean simulations*

Comment 5

The authors applied their definition of hybridness to many gene network models. While this is useful, it is unclear how one can apply this metric to connect models to experimental data. There is a wealth of published data on progression of EMT in cell lines and EMP-related tumors. It will be helpful to show that the authors' analysis of hybridness can elucidate hybrid cells/samples in data.

Response:

We thank the reviewer for highlighting this important aspect. We are currently bench-marking in various experimental datasets of the EMT scoring and hybridness calculated using the metrics shown in this manuscript vs. the previously defined EMT scoring ones with a large gene count (Tan *et al.* EMBO Mol Med 2014, doi: 10.15252/emmm.201404208; Byers *et al.* Cancer Res 2013, doi: 10.1158/1078-0432.CCR-12-1558). This characterization effort is an extensive one, thus beyond the scope of this manuscript. We plan to include this analysis in an upcoming manuscript.

Comment 6

The authors provided a good discussion on the trade-off between hybridness and multistability. However, some additional reconciliation between the authors' findings on hybridness and previous theories of hybrid E/M states (including the authors' own work doi: 10.1073/pnas.1318192110) seems to be necessary. In previous models, hybrid E/M states occur in conjunction with multistability, but the authors claimed an anticorrelation between hybridness and multistability in the present work. This discrepancy can be confusing to readers if the authors do not provide a clearer explanation.

Response:

We have now expanded upon the discussion to clear the possible confusion. The updated text is given here;

"A particularly interesting observation is the negative correlation between hybridness and multistability. While it may appear counter-intuitive, given that cells showing hybrid phenotypes have been shown to be highly plastic (i.e., switch readily to other phenotypes), a closer look can resolve this apparent contradiction. The high plasticity of hybrid phenotypes indicates their lesser stability as compared to Epithelial and Mesenchymal phenotypes, an observation that has been reflected in our simulations of the wild-type EMT networks as well.

In other words, for wild-type networks in the parameter regimes where multistability is allowed, the fraction of initial conditions converging to hybrid phenotypes will be much less as compared to the fraction of initial conditions converging to Epithelial or Mesenchymal phenotypes. On the other hand, the negative correlation between multistability and hybridness occurs as perturbations are made to the network topology that increases the stability of hybrid phenotypes (thereby decreasing the stability of the Epithelial and Mesenchymal phenotypes)."

<u>Comment 7</u>

Please fix some minor issued in figures, e.g. the label 'Percentile' overlapped with a number in the color bar of Figure 7E, and an incomplete self-regulation arrow of OVOL2 in Figure S1C.

Response:

We thank the reviewer for highlighting these issues that have been fixed now.

Reviewer #2:

The manuscript describes an interesting and extensive analysis of EMP networks, important in advancing our understanding of metastasis. Overall, I enjoyed reading the manuscript but there are many places where things are described inaccurately or wrong, not supported conclusions are drawn. Below my major concerns, followed by minor concerns and some selected language issues (there are many more I did not point out).

It seems to me that the intro and discussion was carefully edited, probably by the senior author, but the same level of care lacks in the rest of the manuscript (especially, methods and results description). One example: Many of the math formulas are written in poor, inaccurate notation. Mostly, I assume I get what is meant but cannot be sure. I really would like the authors to take more time and clearly define everything needed to accurately describe the different measures. It may require a few more lines of definitions but it will be a great service to anyone who really wants to builds upon this work or check it. For example, in the formula of frustration of a state F_S, the denominator seems wrong. If sign(J_ij) in {-1,1}, then the sum of this is not the total number of edges involved in that state as stated just below. Moreover, the mathematical notation of the two sums is confusing. Why is the sum taken over S? That doesn't seem right/accurate. The next formula for F_N is also inaccurate, and so is the formula for (), etc.

We are grateful to the reviewer for taking time in reading our manuscript thoroughly and providing many helpful suggestions/comments that have considerably improved our manuscript.

We have revised the formula for frustration. The updated text is as below;

"For a given state, if the configuration of two nodes (S_i, S_j) conflicts with the edge connecting the two nodes (J_{ij}), the edge is considered frustrated for that state. Hence, a frustrated edge would satisfy:

$$sign(J_{ij}).sign(S_i).sign(S_j) = -1$$

The frustration of a steady state (F_{SS}) is calculated as the number of frustrated edges (F_J) in that state divided by the total number of edges (E) in the network.

$$F_{SS} = \frac{F_J}{E}$$

We then estimated network-level frustration (F_N) using the following three metrics: minimum, maximum, and mean frustration, and compared these against the hybridness of the networks."

$$F_N = \begin{cases} Min(F_{SS}), SS\ spans\ all\ the\ steady\ states \\ Max(F_{SS}), SS\ spans\ all\ the\ steady\ states \\ Mean(F_{SS}), SS\ spans\ all\ the\ steady\ states \end{cases}$$

Some conclusions in the manuscript are much too strongly worded and/or the way statistics is used to support the claim is questionable. Here are some examples:

<u>Comment 1</u>

Abstract: The abstract may be a little misleading. The authors write: "We found that increasing negative feedback loops enhances the "hybridness" of these networks, but increasing the number of positive feedback loops or their specific combinations can decrease the frequency of hybrid E/M phenotype." 1. The study only shows that there is an association between many negative FBLs and hybridness, not that a given network can gain more hybridness by adding more negative FBLs.

Response:

Thank you for this suggestion. We have modified the abstract and the updated text is given below;

"…Here, we investigate the dynamics of 13 different GRNs and report an interesting association between "hybridness" and the number of negative/positive feedback loops across the networks. While networks having more negative feedback loops favor hybrid phenotype(s), networks having more positive feedback loops (PFLs) or many HiLoops – specific combinations of PFLs, support terminal (E and M) phenotypes. We also establish a connection between "hybridness" and network-frustration by showing that hybrid phenotypes likely result from non-reinforcing interactions among network nodes (genes) and therefore tend to be more frustrated (less stable)."

<u>Comment 2</u>

"Our results show that WT networks are highly multistable compared to their perturbed counterparts (Fig 3B)". The results shown in Fig 3B support the statement "WT networks are highly multi stable". However, only for the 2 out of 13 networks that are shown here. What about the 11 others? The latter part "compared to their perturbed counterparts" is not sufficiently supported. The perturbed networks can be considered as null models. A standard way to analyze network properties using null models is to (i) compute a certain property for the original networks and then (ii) use a "shuffled p value" comparing these properties (i.e., the value for the original network and the distribution of null model values). If $p\_shuffle<0.05$, which it clearly isn't in Fig 3B, one could argue that this finding is not just due to chance.

Response:

The scatter plots for 11 other networks have now been included as **Fig S4**. We are thankful for the reviewer's suggestion of calculating a shuffled-p value for multistability. In the results given below **(Fig R2)**, we found that the p-value is greater than 0.25 for 12 out of 13 networks, suggesting that the WT networks do not have a significantly high incidence of multistability.

However, the negative correlation between Hybridness and Multistability (Fig 3C) is consistently negative, irrespective of the observation in shuffled p-value above. Hence, we have remove the statement saying that WT networks have higher multistability than perturbed networks from the manuscript, as it is of no consequence.

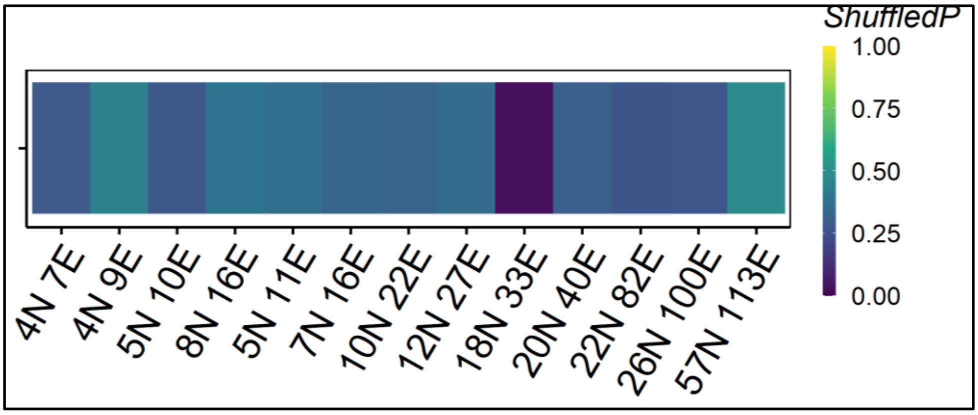

Figure R2. *Heat map showing shuffled P value cross EMP networks.*

<u>Comment 3</u>

“while weighted PFLs showed weaker correlation with hybridness as compared to their un-weighted counterpart, the weighted NFLs led to improved correlation as compared to un-weighted NFLs (10/13 significant)”. The authors compute the correlation of hybridness with various measures and use the proportion out of the 13 networks for which p<0.05 as a means to derive “improved correlation”. Any statistician would turn their head at this practice. Looking at the actual 13 correlation values, e.g. for weighted # of NFLs (with hybridness) and unweighted # of NFLs (with hybridness), one sees that all 13 correlation values almost exact coincide (Fig 5B). The same is true for PFLs and Fraction of PFLs so there is no difference whatsoever and the authors’ conclusions are misleading. Hierarchical clustering of the various compared measures based on their correlations with hybridness across the 13 networks would help to see this close similarity, and would also be a useful method for other similar plots.

Response:

We agree with the reviewer that there is very little difference in the correlation strengths for weighted and unweighted NFLs (this gets clearer with the clustered heatmaps suggested by the reviewer). The point we were trying to make was about the consistency of these correlations being higher for weighted NFLs as compared to the unweighted NFLs, given that the correlations are being calculated over the same number of observations for a given network.

<u>Comment 4</u>

Everywhere, the authors compute the Spearman correlation between hybridness and various measures. However, for the different 13 networks, the number of observations (i.e., null

models) seems to equal the number of edges. When using p<0.05 as a means to differentiate significance this introduces a bias against networks with fewer edges as a higher correlation value is needed to achieve p<0.05. It's a classical example of the over-use of p, rather than relying on the effect sizes (here, the correlation values). This (among many other papers) may be a useful read for some of the junior authors: Kim, Jeehyoung, and Heejung Bang. "Three common misuses of P values." Dental hypotheses 7, no. 3 (2016): 73.

Response:

We thank the reviewer for providing the useful reference on p-value. Our data in almost every network is monotonic (due to outliers). Since, Spearman correlation gives better prediction of p value than Pearson correlation for such type of data where the assumption of linearity does not hold, that's why we used Spearman correlation. We also understand that it could introduce bias in smaller networks due to small data size, however, we don't think other methods of calculating and interpreting p-value can help in this particular data scenario.

Some of these shortcomings described above are to be expected in a study like this. For instance, it is not clear how to create the same number of null models for all 13 networks given different numbers of edges. However, I really missed in the Discussion a paragraph or two addressing the limitations and shortcomings of this study. This should not just include the few things I pointed out but the authors, who are the experts in this field, should brainstorm and describe possible confounders/biases in their study.

Response:

We have now extended the last paragraph to include the shortcomings of the study. The updated text is below;

"…we however report a few shortfalls of the study: (1) the analysis is of correlate nature and lacks a thorough causative investigation. (2) Compared to larger networks, the smaller number of perturbed cases for smaller networks raises a possibility of misinterpretation of p-value significance there. (3) Third, the 'hybdriness' feature needs to be compared against transcriptomic measures of hybrid E/M phenotypes."

Minor comments:

<u>Comment 1</u>

P3: results, first paragraph: The authors should state what kind of models the 13 EMP GRNs are, or are they simply wiring diagram without an underlying model describing the update rules (e.g., Boolean functions or differential equations)? Also, citations to the <= 13 papers the 13 EMP GRN models come from wouldn't hurt here.

Response:

We have now added references to all the models and modified the language as well. The models have been constructed from the experimental data and have previously been reported in the references cited in the updated text below;

*"To understand the emergence and frequency of hybrid phenotypes, we investigated 13 different GRNs of varying sizes. These networks can be categorized into three classes based on their size (number of nodes and edges): (i) four small-sized networks (**Fig 1A i, Fig S1**). (ii) four composite networks formed by combining the small-sized networks (**Fig 1A ii, Fig S1)** and (iii) five large-scale networks (**Fig 1A iii, Fig S1**). We labelled each network with the number of nodes and edges in the network (ex: 4N 7E for the smallest network). The small and large networks have previously been reported to be underlying EMP (Jolly et al. 2016; Boci et al 2019; Silveira and Mombach, 2019; Silveira et al. 2020; Huang et al. 2017; Tripathi et al. 2020a; Font-Clos et al. 2018; Hari et al. 2020; Hari et al. 20212). Given the demonstrated role of these networks in EMP, we constructed the composite networks to check the validity of our results when the assumption that these networks are isolated is relaxed.*

While most of these networks had previously been simulated with a specific set of kinetic parameters/Boolean rules, we used only the topologies for our analysis to understand the effect of topology on emergent hybridness."

<u>Comment 2</u>

P3: Formula: The authors write "If the number of activators incident on the node is greater than the number of inhibitors, node is set to 1, otherwise 0." But in the formula the case sum = 0 is excluded. From my understanding of threshold rules, if sum = 0, then the gene remains at its current value. If that is what the authors used here, it needs to be described as this case happens frequently with |J_ij| = 1

Response:

Thank you for bringing it to our notice. The Boolean function has been revised.

$$S_i(t+1) = \begin{cases} 1, & if\ sign\left(\sum_j J_{ij}\, S_j(t)\right) > 0 \\ 0, & if\ sign\left(\sum_j J_{ij}\, S_j(t)\right) < 0 \\ S_i(t), & otherwise \end{cases}$$

where, J_{ij}, the edge from the node S_j to node S_i, is defined as,

$$J_{ij} = \begin{cases} 1, & if\ S_j\ activates\ S_i \\ -1, & if\ S_j\ inhibits\ S_i \end{cases}$$

Comment 3

P3/P4: The definition of hybridness is not clear to me: Here the authors write "define the 'hybridness' of a network as fraction of steady state frequencies that can be classified as hybrid E/M". Frequency should be explained. For Boolean networks, the frequency of a steady state corresponds to the proportion of all 2^N states that eventually end up in this steady state (i.e., the proportional basin size). In the asynchronous update scheme, think of the BN as a Markov chain. From this Markov chain with transition matrix A of size 2^N x 2^N one can read off the basin sizes from the final transition matrix A^∞. The problem with the definition of hybridness is two-fold:

(1). In Fig 1C, it is defined as the sum of all frequencies of hybrid steady states and Fig 1D shows values in [0,1], which makes sense. However, in the methods it is defined as the sum of all frequencies of hybrid steady states over the sum of all frequencies of all stable steady states (where stable is by the way a terrible choice word (every steady state in a BN is stable), how about terminal?). This latter definition would imply values in (0,infinity) and could even lead to issues if there are no "stable" steady states at all. I think the first definition is what the authors should use, it is much simpler and straight forward.

Response:

We are sorry for this inconsistency in hybridness definition. We have made changes in the methods section by sticking to the definition as in Fig. 1D. So, as suggested by the reviewer, we simply define hybridness as the sum of frequencies of hybrid steady states.

(2). If using the first definition, one question remains: What about limit cycles? For a general Boolean network, some attractors are steady states but most are typically limit cycles, meaning on average most of the 2^N states may actually end up in a limit cycle. Are they simply ignored? Can the authors prove that there are no limit cycles for the 13 networks they consider? The latter is a possibility given that the authors consider an asynchronous update scheme.

Response:

As mentioned in the methods section, we considered an asynchronous update scheme.

"The simulations were performed asynchronously, i.e., at each time step, one node in the network is randomly chosen and updated. Each network was simulated for 100,000 initial conditions, with a maximum of 1000 time steps per initial condition. All simulations were repeated three times."

To further verify that the most of the attractors in these networks are actually steady states, we simulated each WT and perturbed network for 1,000,000 initial conditions (now 10 times more than in previous simulations). Each initial condition is simulated for 1000 time steps to track if the network reaches a steady state. The fraction of initial conditions for which a steady state has NOT been reached (indicating a random fluctuation or a limit cycle) is reported as the frequency of non-steady states. We didn’t find the possibility of limit cycle in 11 out of 13 networks in either WT (Fig R3) or perturbed networks (Fig R4). Also, in the remaining three networks the frequency of a limit cycle seems to be very less.

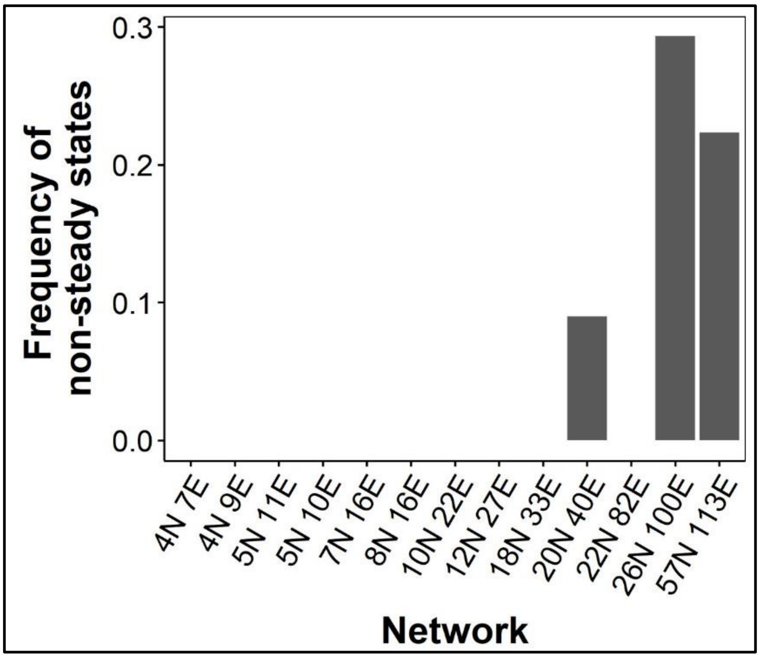

Figure R3. Frequency of non-steady states in wild type EMP networks

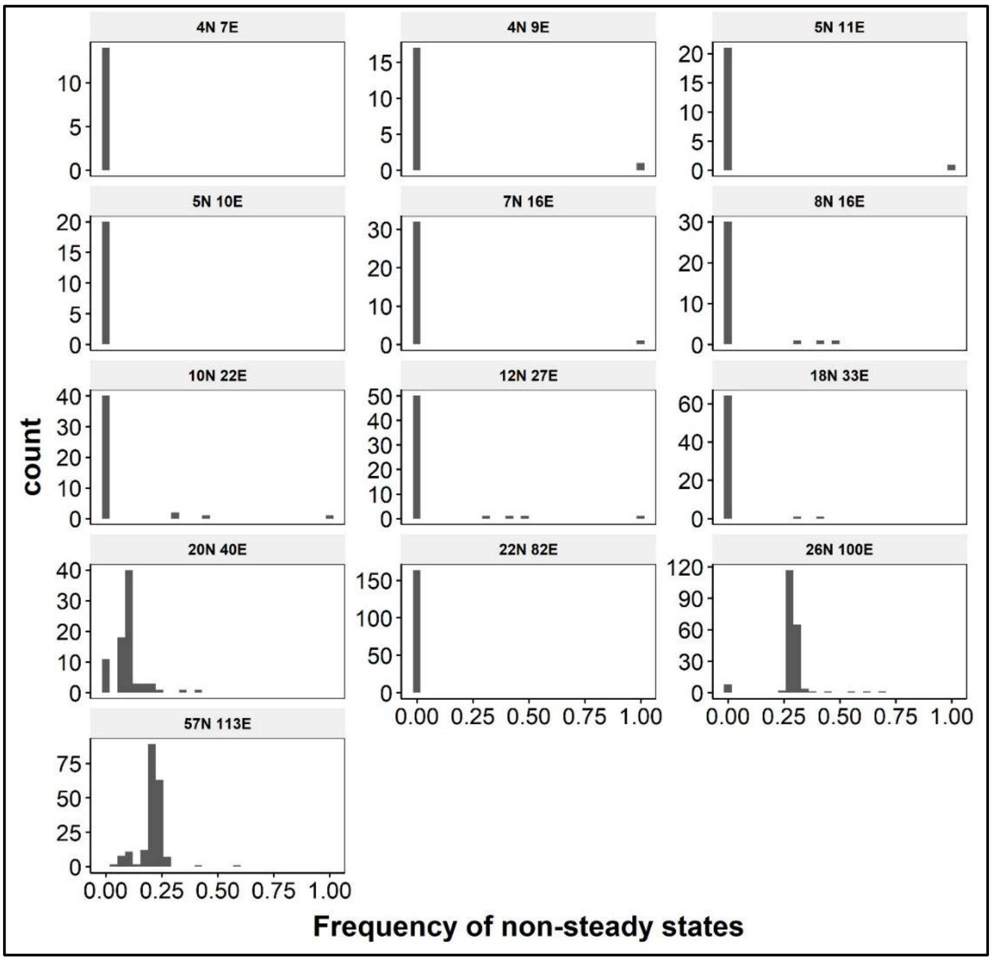

Figure R4. Frequency of non-steady states in perturbed EMP networks

Comment 4

There are two figures labelled Figure 1. This needs to be fixed. The figures are also not labelled in order. Figure 5 appears after figure 6, etc.

Response:

We are sorry for this mistake. The figure numbers have now been corrected.

Comment 5

I know the EMT score has already been published. I have two concerns regarding the way it is used here nevertheless. 1. Defining a hybrid state as having |EMT|<0.5 means smaller models will on average have a smaller proportion of hybrid states because |EMT|<0.5 does not scale with network size. 2. What about the relative number of ENodes vs MNodes. If there is many more of type than the other, as in e.g. 57N 113E, then by random expectation, many

states are classified as hybrid. I suggest two possible modifications: Account for network size and account for the relative size of Enodes vs MNodes.

Response:

We have accounted for the relative size of E nodes vs M nodes while calculating the EMT score. The formula mistakenly did not include this part previously and has now been rectified. I don’t see how it does not scale with network size. The E score and M score of a state are defined with the number of E and M nodes respectively as the normalizing factor, which should take care of the network size bias?

<u>Comment 6</u>

P5: The sentence where Fig 2B is referenced does not seem to really refer to what is shown in Fig 2B. What is shown here, is not explained anywhere in the text.

Response:

We have made necessary changes in the manuscript. The edge perturbation indices (1 to 14) in the x-axis of Fig 2B are actually explained in Table S1 in the supporting information. With Table S1, things should make sense.

<u>Comment 7</u>

P5: J metric formula, why mod(a_ij) simply a_ij with a_ij already being described as the correlation between i and j?. Also “where a_ij is the any element…”, delete the

Response:

We apologize for the mistake. We have removed mod from the formula.

<u>Comment 8</u>

P8: Formula of multistability: this is sloppy notation. It should be $\sum_{P_MS} 1$ or simply define p_MS as the proportion of parameter sets that give rise to multiple stable states and use this as the measure of multistability.

Response:

We feel the notation is unnecessary there and have removed it now. As suggested, we simply defined multistability as the proportion of parameter sets that give rise to multiple steady states.

Comment 9

P10: “Fig 5A, i-iii” and “Fig 5A, iv-vi”, there are no such sub labels in the figure.

Response:

These labels have been added.

Comment 10

P10: “Given that the shorter loops are likely to be more effective”. More effective in what? This needs to be added.

Response:

We have deleted this sentence.

Comment 11

P10: Positive Loops (Influence) is a bad description of the measure. How about Number of Node Pairs with coherent influence, or short, Number of coherent node pairs because really you are not looking at loops in this measure but at pairs of nodes (in a way, 2-loops).

Response:

With this description we just want to highlight that the influence of the nodes in the loops is also taken care of in this measure. Moreover, we want to restrict to this notation as there is a possibility that the suggested notation “incoherent node pairs” for “negative loops (influence)” may create confusion with the “inconsistency” measure calculated in the later part of the study.

Comment 12

There is a grey unexplained cell in Fig 6D.

Response:

We don’t have any Type 2 loop in either 4N 7E network or its perturbations that is why the grey cell is there. We have included this explanation in the figure caption as well.

Comment 13

The description of inconsistency is not accurate enough, in the main text and in the methods section “Inconsistency”. For example, inconsistent feed-forward loops are not at all explained (a reference e.g. to Uri Alon’s work may suffice). Further, the sentence “Inconsistent connections include those involved in negative feedback loops and inconsistent feed-forward loops (estimated by counting the number of un-directed negative loops)” does not provide enough information to really understand what exactly is computed here.

Response:

We have made the changes in the main text and briefly introduced the definition of inconsistency. We also provided more explanation of inconsistency in methods section.

“The inconsistent feedback/feedforward loops are those in which different paths between any two nodes contradict rather than reinforce. For example, negative feedback loops are inconsistent while as positive feedback loops are consistent. We define inconsistency (or consistency deficit) of a network as the minimum number of edges that must be removed or whose signs must be flipped so that the network becomes consistent. The inconsistency of the network is estimated using the following steps. We first use NetworkX package in python to calculate the number of undirected loops in a network. Then, we find out the common edges across the identified loops and iterate through the ones common between negative loops in the order of the number of loops they are common in and flip them one-by-one until all negative loops turn positive. The number of flips that are required to achieve this is labelled as inconsistency of a network.”

Comment 14

P19: what does triplicate mean here. What exactly was repeated three times?

Response:

We apologize for the confusion, and have modified the language in the methods section. The updated text is given here;

“Each network is simulated with $10{,}000$ random parameter sets and each simulation is repeated three times to reduce the chance of parameter bias. We took the average of the three simulations and generated $10{,}000$ systems of ODE’s for each network, where each system has as many equations as the number of nodes in the network.”

Comment 15

Figure 5 (should be 7) legend: X: p-value > 0.05 should go with B.

Response:

We have made the necessary changes.

Selected language issues:

P2: it should be "Recent efforts have identifIED some individual factors"

p2: 4nodes/7edges, spaces missing

P3: "can significantly enhances", enhance not enhances

P3: subsection header: changes not change, also rather than changes with changing network-topology, I would say "Hybridness" of EMP networks is associated with network topology, also no dash, or network topology affects "Hybridness" of EMP networks.

Methods: Delete this unnecessary, awkward sentence: Likewise, the method follows in all the other networks.

P7: also called as phenotypic plasticity, delete as

P7: It has been observed both in vitro as well as in vivo studies, delete studies

P7: replace "as compared to the" by "than"

P9: figure legend: Spearman must be capitalized. On the other hand, I would never capitalize hybridness, mean frustration, maximum fr., minimum fr., etc.

P10: replace "potentially of saturation" by "potentially due to saturation"

P10: delete as in: and called it as "predicted frustration"

P10: "the total number of negative feedback loops (NFLs) WAS"

P11: "takes into accounts", delete the s

Response:

We can't thank reviewer #2 enough for highlighting these mistakes. All these corrections have been fixed.

Reviewer #3:

In this work, the authors used the networks underlying mathematical models of EMT to study the relationship between network topology metrics and dynamics-based measures (particularly “hybridness”) across an ensemble of networks. The ensemble of networks studied were all single-edge perturbed networks w.r.t to the original network. Among the network topology measures explored are measured based on feedback loops (positive/negative, weighted/unweighted, influence-based, special types of loops). Among the dynamics-based measures explored are hybridness, frustration, multi-stability, and global expression patterns (JSD, J).

The networks were based on previously published models or their combination and ranged from 4 nodes and 7 edges to ~60 nodes and ~100 edges. For the hybridness and dynamics-based measures, they used ensembles of models with fixed network topology, leveraging methods they or others previously developed (RACIPE, Font-Clos et al. 2018, and others).

Among their main findings are (1) that the original networks have a lower hybridness than the single-edged-perturbed networks, (2) that the global expression patterns correlate with hybridness, (3) that multi-stability is inversely correlated with hybridness, (4) that hybrid phenotypes have high values of frustration, and (5) that positive/negative feedback loops are negatively/positively correlated with hybridness.

Overall, I find the study interesting because it is studying the relationship between topological measures and dynamics-based measures using networks from EMT models. However, my takeaway from the results is that most of these correlations where what we were expecting from the types of networks being studied (almost-sign-coherent networks that can support intermediate states) + the way the dynamic measures are defined (e.g. hybridness based on clustering of steady states + hybrid score from marker nodes). The authors are also primarily leveraging the tools and approaches they and others have developed to study a new ensemble of networks (single-edged-perturbed networks). In other words, I do not see “Significant biological and/or methodological insight” or “High importance to researchers in the field” (following the PLOS Computational Biology criteria). In addition, I have concerns about how much is being learned about EMT per se vs generic properties of biological networks that support hybrid states. In my view, this study seems to be a better fit for a more math-oriented or physics-oriented journal, rather than a biology or computation biology journal. This is particularly true because no biological data is used besides the EMT networks, and there are no restrictions to the parameters explored to have biologically realistic behaviors. There is also the additional problem that most of the analyses do not have a clear “negative control” (e.g. the fully rewired networks explored in the last part) or “positive control”. This is particularly important because the bulk of the work is based on correlations from complex processed data (dynamics + z-scores + ensembles + clustering + hybrid scores) with variables that explain a small amount of the variance (even if statistically significant) and often have multiple outliers.

We thank the reviewer for showing interest in our work. Our study particularly highlights association of network topologies with the likeliness of hybridness in EMT networks. Previous computational studies have reported that the hybrid phenotypes can occur by “tuning” the expressions of specific molecules (called as phenotypic stability factors). We however report that the fashion (or the topology) in which the genes in the EMT networks

interact can also favour the emergence of hybrid phenotypes, thus providing an additional perspective on the source of emergence of the non-genetic tumor heterogeneity. Further, an important aspect of our study is that regardless of the size (node and edge numbers) of these EMP networks our results show consistency across.

Other comments:

Comment 1

The authors should include a more comprehensive analysis of the consistency checks used to make sure the clustering steps used to obtain the E and M genes for each network. The authors report that they verified that the expected E and M genes are obtained with this analysis, but it is not clear exactly what this means. E.g. some heatmaps with information on their E/M/Hybrid classification, the E and M nodes, and the frequencies used for the hybridness calculations for each network would be helpful.

Response:

We have now added a section on "classification of network nodes" in methods where all the steps are clustering steps are discussed. The updated text is below;

"Classification of network nodes
Given the nature of the networks analyzed here, we could classify the nodes (genes) participating in the network into two categories: Epithelial and Mesenchymal. First, we calculated pairwise correlations between the expression values of the nodes obtained from RACIPE, using the cor function, thus obtaining a correlation matrix. We then applied hierarchical clustering on the correlation matrix (hclust, distance method Euclidean, agglomeration method complete). We split the resultant dendrogram into two chunks (cutree), resulting in two clusters of nodes. These clusters were then labelled based on the biochemical nature of the composing nodes as Epithelial or Mesenchymal."

Comment 2

Why is there no exploration between multi-stability and hybridness in Boolean systems?

Response:

We thank the reviewer for the question. The reason we did not investigate this aspect in Boolean systems is the difficulty in defining multistability for Boolean simulations. For RACIPE, the usage of ensemble of kinetic parameter space leads to a clear identification of parameter sets that allow multistability. Thus, each unique parameter set in RACIPE can be classified as multistable or not. From a biological perspective, each parameter set in RACIPE represents a cell in a heterogeneous population, and multistability refers to the fraction of cells in the population that can access multiple phenotypes, when starting from different

initial conditions. However, as Boolean simulations use no kinetic parameter sets, defining multistable or not for a given iteration of Boolean simulation becomes infeasible.

<u>Comment 3</u>

Frustration is defined based on all states, ignoring the fact that each is associated to a parameter set. This seems to be different to how the other metrics dynamics-based metrics are defined, and it is not clear to me why. It seems to me that If we looked per parameter set, the EMT score and frustration would be highly correlated (higher near EMT score = 0). By construction of the EMT score and hybridness (through clustering of states), I expect hybrid states near EMT score = 0 to always be highly frustrated. Things get more complicated when looking at summary statistics of frustration values because we are then comparing across parameter sets.

Response:

We apologize for the confusion caused. Here, frustration is defined for an individual, discretized state, similar to the EMT score. Hybridness on the other hand, is defined for the network across a large set of parameters sampled by RACIPE. Hence, to compare frustration with hybridness, we had to use mean, minimum and maximum frustration values across all steady states across all parameter sets.

Additional technical comments

<u>Comment 1</u>

1) EMT score. This seems to be based on prior work by the authors, but it is not clear how it is exactly applied to the case at hand. Do they define a new set of E_nodes and M_nodes for each network presented? Do they do the clustering for each network or is it based on those on the original networks? Do they use all the steady states generated for all parameters set to do this clustering? Isn't this metric inadequate when the size of E_nodes and M_nodes are very different (e.g. the 57N 113E network) or is there some extra normalization in the sum so that it EMT score can be 1 or -1 if the appropriate nodes are active/inactive?

Response:

We classify E and M nodes as explained in the newly introduced section "classification of network nodes" in methods that results into E and M gene sets. Though the process is repeated for each wild-type (WT) network, but it never happens that a gene in one WT network that is classified say as E gene would be classified as M gene in another network.

Also, we apologize for mistake in the EMT score formula. The updated formula is as below and it does take care of relative size of E and M nodes in each network.

$$EMT\ Score = \frac{\sum_{i\,\in Enodes} S_i}{\#\ of\ Enodes} - \frac{\sum_{j\,\in Mnodes} S_j}{\#\ of\ Mnodes}$$

Comment 2

2) Hybridness. Is the frequency of hybrid states based on total number of steady states, regardless of how often they appear per parameter set? The methods say “frequencies of the fraction of steady state frequencies that are hybrid” which I assume means “per fixed parameter set, fraction of steady states that are hybrid”, but the way it is phrased does not make it clear to me. E.g. if few parameter sets give 100 hybrid states, and all other parameter sets give 2 terminal states, hybridness could be very high or low depending on how it is calculated.

Response:

Sorry for this confusion. We have updated the definitions of EMT score and hybridness in Fig. 1C and in the corresponding caption. A more description can be found in “Hybridness Calculation” in methods section.

To explain it briefly with an example here, we define hybridness of a network as the sum total frequencies of all the hybrid states. These frequencies are already normalized by the total frequency count of all the steady states of a network. For example, suppose a network is simulated for 1000 parameter sets. Suppose after binarization of steady states (SS), we find that there are only 10 unique SS and each SS is repeated 150 times. Well, it can happen that the total repetitions of SS (1500 in this case) are more than total number of parameter sets (1000 in this case), since we are solving each model for 100 initial conditions and some models could be bistable, tristable, etc. Then, 1500 (=10x150) is the total SS frequency of the network. After classifying these SS into E, M, and hybrid states based on the EMT score, suppose we find that only 5 out of the 10 SS are hybrid. Since each of the hybrid SS is also repeated 150 times, the total hybrid frequency would be then 750 (=5x150). The hybridness of the network would be then 0.5 (=750/1500).

Minor comments:

Comment 1

The figure numbers are wrong. There are two figure 1.

Response:

We have corrected the figure numbers.

Comment 2

The authors do not specify the references from which they obtained the EMT networks.

Response:

The references are now added in the updated text given below;

“The small and large networks have previously been reported to be underlying EMP (Jolly et al. 2016; Boci et al 2019; Silveira & Mombach, 2019; Silveira et al. 2020; Huang et al. 2017; Tripathi et al. 2020a; Font-Clos et al. 2018; Hari et al. 2020; Hari et al. 2021). Given the demonstrated role of these networks in EMP, we constructed the composite networks to check the validity of our results when the assumption that these networks are isolated is relaxed.”

<u>Comment 3</u>

There is Inconsistency between statistical significance in the plots. E.g. S3A 22N 82E has an asterisk showing significance, but an X in S3C showing lack of significance. Similarly for S3B 22N 82E.

Response:

We apologize for the mistake. We have fixed these errors and updated the figure.
